# Lead-Free Sodium Potassium Niobate-Based Multilayer Structures for Ultrasound Transducer Applications

**DOI:** 10.3390/s22093223

**Published:** 2022-04-22

**Authors:** Danjela Kuscer, Brigita Kmet, Silvo Drnovšek, Julien Bustillo, Franck Levassort

**Affiliations:** 1 Jožef Stefan Institute, Jamova cesta 39, 1000 Ljubljana, Slovenia; brigita.kmet@ijs.si (B.K.); silvo.drnovsek@ijs.si (S.D.); 2 GREMAN UMR 7347, Université de Tours, CNRS, INSA CVL, 16 rue Pierre et Marie Curie, CEDEX 2, 37071 Tours, France; julien.bustillo@insa-cvl.fr (J.B.); franck.levassort@univ-tours.fr (F.L.)

**Keywords:** KNN, thick films, screen printing, ceramic backing, sacrificial template, microstructure, sintering, acoustic attenuation, ultrasound transducers, imaging

## Abstract

Thick films with nominal composition (K_0_._5_Na_0_._5_)_0_._99_Sr_0_._005_NbO_3_ (KNNSr) on porous ceramics with identical nominal composition were investigated as potential candidates for environmentally benign ultrasonic transducers composed entirely of inorganic materials. In this paper, the processing of the multilayer structure, namely, the thick film by screen printing and the porous ceramic by sacrificial template method, is related to their phase composition, microstructure, electromechanical, and acoustic properties to understand the performance of the devices. The ceramic with a homogeneous distribution of 8 μm pores had a sufficiently high attenuation coefficient of 0.5 dB/mm/MHz and served as an effective backing. The KNNSr thick films sintered at 1100 °C exhibited a homogeneous microstructure and a relative density of 97%, contributing to a large dielectric permittivity and elastic constant and yielding a thickness coupling factor *k_t_* of ~30%. The electroacoustic response of the multilayer structure in water provides a centre frequency of 15 MHz and a very large fractional bandwidth (BW) of 127% at −6 dB. The multilayer structure is a candidate for imaging applications operating above 15 MHz, especially by realising focused-beam structure through lenses to further increase the sensitivity in the focal zone.

## 1. Introduction

Potassium sodium niobate K_0_._5_Na_0_._5_NbO_3_ (KNN)-based materials have been described as environmentally benign piezoelectrics, with a high Curie temperature of ~400 °C, a thickness coupling coefficient *k_t_* of 40–50%, and a moderate dielectric permittivity of several hundred. These properties offer the possibility of integrating KNN-based materials into various electromechanical devices, such as ultrasound transducers (UT), thus reducing the use of toxic lead-containing compositions in electronics [1,2].

For medical imaging, UTs operating in the MHz frequency range require a wide bandwidth (BW), which allows fine axial resolution and a narrow lateral beam width, which is achieved by focusing the beam with a lens or directly with a geometrically shaped piezoelectric element. The piezoelectric has to be a few tens of micrometres thick and integrated on a highly damping substrate. A piezoelectric with a moderate dielectric permittivity allows good electrical matching to the electronics, its high electromechanical coefficients are reflected in a high sensitivity of the single-element transducer, and the pores introduced into the piezoelectric reduce its acoustic impedance and thus improve the acoustic matching of the piezoelectric to the medium under investigation, i.e., water with Z of 1.5 MRa [3,4].

Pores in the ceramic backing also increase the acoustic attenuation coefficient (α), but the pore size and pore size distribution must be carefully designed [5,6,7]. For example, a lead zirconate titanate (PZT) ceramic prepared by mixing the ceramic powder with an organic template, i.e., ammonium oxalate, and then sintering had a relative density of 83%, a non-uniform pore size, and a wide pore size distribution below ~10 µm. α was 0.26 dBmm^−1^ MHz^−1^ [5]. For a PZT ceramic with similar relative density but with homogeneously distributed spherical pores with a similar size of ~10 µm, the value increased significantly to 1.8 dBmm^−1^ MHz^−1^. This ceramic was processed by the sacrificial template method from the suspension based on the hetero coagulation (HC) process of the oppositely charged PZT and polymethyl metacrylate (PMMA) template particles in water followed by sintering. The most important aspect of this process is that the oppositely charged ceramic particles and the template are in the same pH range to achieve homogeneous distribution of both particle types in the powder and subsequently in the ceramic [7,8].

Most KNN-based transducers operating over 15 MHz have been fabricated in the conventional way by machining piezoelectric ceramics into disks a few tens of micrometers thick and then gluing them to a backing based on epoxy resin mixed with metal particles, such as silver or tungsten [9,10]. However, manipulation of such samples is technically difficult and rather time-consuming. An alternative approach for KNN-based UT using thick film technology that allows processing of piezoelectric layers that are tens of micrometers thick directly on the substrate, making the production process simpler and less expensive, was discussed [11,12]. The applicability of the KNN-based transducer for medical imaging was demonstrated. However, little attention has been paid to the chemical composition and structural and microstructural properties of the piezoelectric layer and backing. These data are of utmost importance for understanding and further improving the response of the UT. Our previous work on KNN-based thick films has shown that the processing of the thick film needs to be optimised with respect to sintering in order to achieve the required density and avoid the formation of defects and/or secondary phases that could degrade the functional response [13,14,15,16]. The literature reports that doping KNN with up to 1% Sr inhibits grain growth and improves the density and electrical properties of KNN [17,18].

Here, we report the processing of porous ceramics, which serves as a backing in UT, with nominal composition (K_0_._5_Na_0_._5_)_0_._99_Sr_0_._005_NbO_3_ (KNNSr). We processed the ceramic with the predefined pore size and porosity by the organic template method using the hetero coagulation method followed by sintering. The active piezoelectric layer, KNNSr thick film, was screen printed onto the porous ceramic and fired. The phase composition and microstructure of the KNNSr thick films are discussed and related to their electromechanical properties. We also measured the electroacoustic response of the structure. The high acoustic attenuation coefficient of the backing and the high bandwidth of the structure show the potential of the KNNSr thick films and the porous KNNSr ceramic backing in the miniature UT, which operates above 15 MHz.

## 2. Materials and Methods

Powder with the nominal composition (K_0_._5_Na_0_._5_)_0_._99_Sr_0_._005_NbO_3_ (KNNSr) was prepared from K_2_CO_3_ (anhydrous, 99.9+%, ChemPur, Karlsruhe, Germany), Na_2_CO_3_ (anhydrous, 99.9+%, ChemPur, Karlsruhe, Germany), SrCO_3_ (99,994%, Alfa Aesar, Karlsruhe, Germany), and Nb_2_O_5_ (99.9%, Aldrich, St. Louis, Missouri, MI, USA). The stoichiometric powder mixture was homogenised in a planetary ball mill in acetone for 4 h, dried, and afterwards calcined at 800 °C for 4 h and re-calcined at 750 °C for 4 h with an intermediate milling. Details are described elsewhere [19]. After the calcination, the powder had a perovskite structure and consisted of sub-micrometer-sized particles, with a monomodal particle size distribution with d_v,50_ and d_v,90_ of 0.64 and 1.34 μm, respectively [14].

The porous KNNSr ceramic (KNNSr-C) acting as a backing was prepared by the sacrificial template method from the KNNSr powder and an organic template, i.e., spherical particles of polymethyl methacrylate with median particle sizes of 10 µm (PMMA) (Soken Chemical & Engineering Co., Tokyo, Japan). KNNSr and PMMA were separately homogenised in ultrapure water and mixed together in the volume ratio KNNSr: PMMA of 70:30. KNNSr was stabilised in water with 6 wt.% of polyethyleneimine (PEI, average molecular weight of 10,000, Alfa Aesar, Karlsruhe, Germany) with respect to the solid load, while the PMMA was stabilised electrostatically. Suspension was dried at 105 °C. Powder compacts with a diameter of 12 mm and height of 8 mm were sintered at 1120 °C for 2 h in a flow of synthetic air with heating and cooling rates of 5 °C/min. As a reference, the KNNSr bulk ceramic (bulk) was prepared from the KNNSr powder at identical sintering conditions as KNNSr-C. Details are described in Appendix A.

Onto the top surface of KNNSr-C, the platinum paste (E1192, Ferro Corp., Mayfield Heights, Ohio, OH, USA ) was screen printed and fired at 1100 °C for 1 h with heating and cooling rates of 5 °C/min.

The pastes for the screen printing (KNNSr-paste) were prepared from 60 wt.% of KNNSr powder that was mixed with 40 wt.% of organic vehicle, comprising α-terpineol (≥98%, Merck, Darmstadt, Germany), ethylcellulose (48% ethoxyl, Sigma Aldrich, St. Louis, MO, USA), and [2-(2-butoxi-etoxi-ethyl)] acetate (≥98%, Merck, Darmstadt, Germany).The paste was screen printed (2 passes) onto the platinised KNNSr-C. The samples were isostatically pressed at 300 MPa and subsequently heated to 500 °C for 1 h and then sintered at 1100 °C for 2 h in a flow of oxygen. The heating and cooling rates were 2 °C/min. The test structure is denoted as KNNSr-T. As a reference, the KNNSr-paste was screen printed on a platinised alumina substrate (A394, Kyocera, Kyoto, Japan) and cured at identical conditions as on KNNSr-C. The sample is denoted as KNNSr-AO.

The phase composition of the samples was analysed by X-ray powder diffraction (XRD) at room temperature using a high-resolution PANalytical diffractometer (X’Pert PRO MPD, Almelo, The Netherlands). The data were collected in the 2θ range from 20° to 70° in steps of 0.017°, with an integration time of 200 s. The phases were identified using the software X-Pert High Score and the PDF-2 database [20].

The microstructure of the ceramic and thick films was investigated by a scanning electron microscope (SEM; JSM 7600F, JEOL, Tokyo, Japan) equipped with an energy dispersive X-ray spectroscopy detector (EDXS; INCA Oxford 350 EDS SDD, Oxford Instruments, Abingdon, UK ). Prior to the analysis, the thick films were cut in cross-section orientation, mounted in epoxy resin, grinded by SiC papers, polished by diamond paste, and afterwards coated with a 5 nm-thick carbon layer using PECS 682 (Gatan, Pleasanton, CA, USA).

The geometrical density of the porous ceramic was calculated from the mass and dimensions of the sample. The relative density of the thick films was evaluated from binary images of the original SEM cross-section images using ImageJ software (1.8.0., National Institute of Health, Bethesda, Maryland, MD, USA). 

The thickness of as-deposited and sintered thick films was measured by contact profilometer (Bruker DektakXT Advanced System, Karlsruhe, Germany).

The dynamic sintering curves of the powder compacts were recorded upon heating to 1150 °C in an air atmosphere using an optical dilatometer (Leitz V. 1A, Leitz, Wetzlar, Germany). The dimensions of the powder compacts were continuously measured from digitalised images, and the relative shrinkages (ΔL/L) were determined.

For the electrical characterisation, the top gold electrodes with a thickness of ∼100 nm were deposited on the sintered samples using RF-magnetron sputtering equipment (5 Pascal, Milan, Italy). The electrodes with a diameter of 3 mm were sputtered on KNNSr-T and KNNSr-AO. The relative dielectric permittivity (ε) and dielectric losses (tan δ) of the samples were measured as a function of temperature at 100 kHz with an impedance spectroscopy analyser (4192A Hewlett Packard, Palo Alto, CA, USA). The ε- and tan δ-versus-temperature curves were recorded upon cooling.

The samples were poled with a DC-electric field of 3 kV/mm for 120 min at 120 °C and then cooled to room temperature under an applied electric field. The direct piezoelectric response of the samples was measured with a Berlincourt piezo d_33_ m (Take Control PM10, Birmingham, UK).

Acoustic properties, i.e., longitudinal wave velocity and attenuation of cylinder-shape KNNSr-C ceramic covered with parylene (PDS2000, SCS, Indianapolis, IN, USA), were measured using a transmission method with two commercial transducers (one transmitter and one receiver), with a centre frequency (*f_c_*) at 10 MHz (Olympus, Rungis, France). The details are described in Appendix B.

The electromechanical properties of KNNSr-T and KNNSr-AO were deduced from the measurements of the complex electrical impedance around the fundamental thickness-mode resonance in air (front face) using an HP4395A spectrum analyser (Agilent Technologies Inc., Palo Alto, CA, USA) and its impedance test kit. An equivalent electrical circuit model (KLM) was used to calculate the theoretical complex electrical impedance of multilayer structures [21]. Details are given in Appendix C.

For acoustic characterisation of test structure KNNSr-T, electrical contacts were made with two thin copper wires bonded to the electrodes on the bottom and top faces of the piezoelectric thick film. A test structure was inserted in polymer-based housing. A coaxial cable with a characteristic impedance *Z_c_* = 50 Ω was used for the electrical connection on the rear face to the generator. Finally, a parylene layer with a thickness of <100 nm was deposited on the top face of the test structure to avoid direct contact with water. The test structure was immersed in a water tank in front of a metallic target to measure the electroacoustic response of the transducer. A lab-made pulser/receiver, a digital oscilloscope (LeCroy waverunner 64XI, Charlottesville, VA, USA), and ~30 cm long 50 Ω cable was used to register the electro-acoustic response.

## 3. Results and Discussion

**Figure 1:**
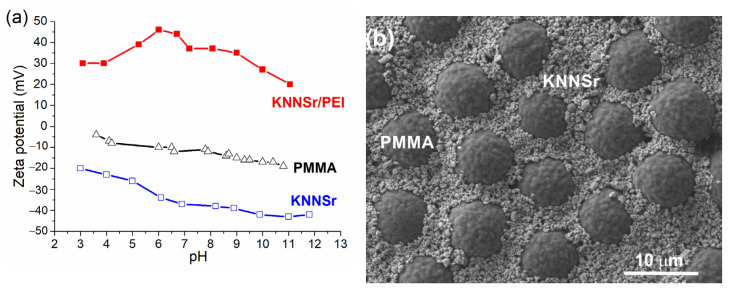
(**a**) Zeta potential (ZP) of the Sr-modified sodium potassium niobate (KNNSr), KNNSr/ polyethylenimine (PEI), and polymethyl methacrylate (PMMA) particles in water as a function of pH; (**b**) scanning electron microscopy (SEM) image of the KNNSr/PMMA powder mixture.

In order to process the powder with a homogeneous distribution of the phases, we investigated the zeta potential (ZP) of PMMA, KNNSr, and KNNSr, stabilised with PEI (KNNSr/PEI) in water as a function of pH (Figure 1a). The ZP of PMMA and KNNSr particles in water was negative in the measured pH range. For PMMA, the absolute value of ZP gradually increases from −5 mV at pH 3.5 to −20 mV at pH 11. The absolute value of ZP for KNNSr is slightly larger with values ranging from −20 mV at pH 3 to −40 mV at pH 12. 

The negative ZP of the individual particles indicates that they repel each other, preventing the hetero coagulation process. Therefore, we stabilised the KNNSr with PEI polyelectrolyte, which resulted in a positive ZP of the KNNSr. The absolute ZP value increased from +30 mV to +48 mV between pH 3 and 6. At higher pH values, the ZP decreases, reaching a ZP of +10 mV at pH 11. The charge reversal of KNNSr upon addition of PEI indicates the adsorption of PEI on the KNNSr surface. PEI with a unit formula of –CH_2_–CH_2_(NH)– is a positively charged electrolyte at low pH. Its degree of dissociation decreases with increasing pH, and at pH 11, it is considered an uncharged polymer [22]. Thus, the decrease in ZP of KNNSr/PEI in the alkaline region is related to the degree of dissociation of PEI.

Based on the ZP measurements, we selected the inherent pH values for processing the suspensions. The inherent pH of 5 vol% KNNSr stabilised with 6 wt% PEI in water was 11.3, and that of 5 vol% PMMA was 6.8. At the selected pH values, the KNNSr particles stabilised with PEI had a positive ZP, while the PMMA particles had a negative ZP. When the two suspensions were mixed in the volume ratio of KNNSr: PMMA of 70:30, the pH was 10.5. At this pH, the ZPs of KNNSr/PEI and PMMA particles had opposite signs, i.e., +25 mV and −17 mV, respectively, and their absolute values were high enough to allow effective dispersion of the particles in water. This enabled an effective HC process, which was reflected in a homogeneous distribution of PMMA and KNNSr/PEI in the powder dried at 105 °C (Figure 1b). The powder obtained is referred to as KNNSr/PMMA.

**Figure 2:**
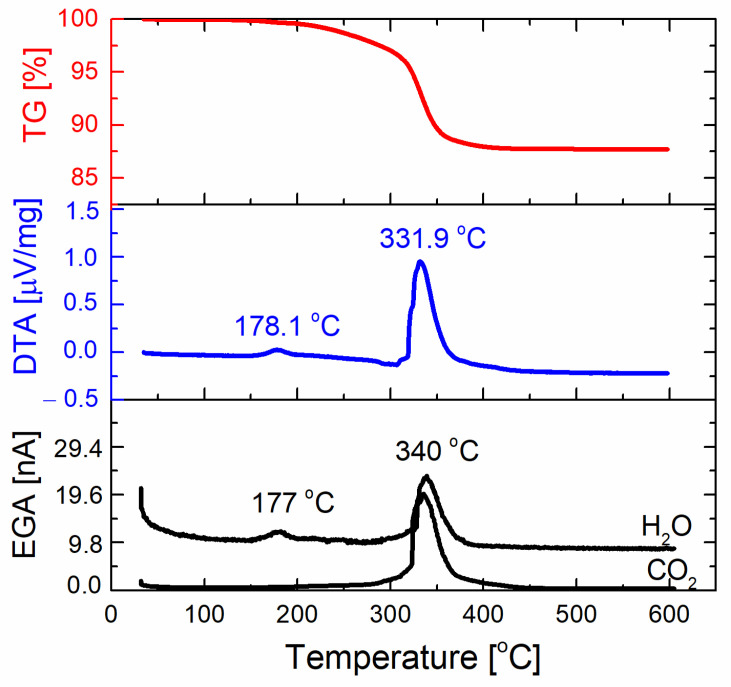
Thermogravimetry (TG), differential thermal analysis (DTA), and evolved-gas analysis (EGA) curves for the KNNSr/PMMA powder mixture with the volume ratio 70:30.

To gain insight into the thermal decomposition of the KNNSr/PMMA powder mixture, thermal analysis of the powder was performed (Figure 2). The total mass loss of the KNNSr: PMMA powder mixture when heated to 400 °C was 12.3%. This mass loss is accompanied by two broad exothermic DTA peaks. The first at 178.1 °C is accompanied by the maximum in the EGA curve corresponding to the release of H_2_O. The second, a stronger peak at 331.9 °C, coinciding with the largest mass loss, corresponds to the release of H_2_O and CO_2_. No further mass loss was observed up to 600 °C. The results prove that complete decomposition of the organic phases occurred up to 400 °C. According to literature, PMMA decomposes in the temperature range between 250 °C and 400 °C [23], while PEI decomposes between 210 °C and 600 °C [24]. The number of organic phases in the mixture of KNNSr and PMMA powders, which was mixed in a volume ratio of 70:30 with the addition of 6 wt.% PEI per mass of KNNSr powder, was 14.6%. The measured mass loss in the powder mixture agrees well with the calculated values and confirms the complete decomposition of the organic phases at up to 400 °C.

**Figure 3:**
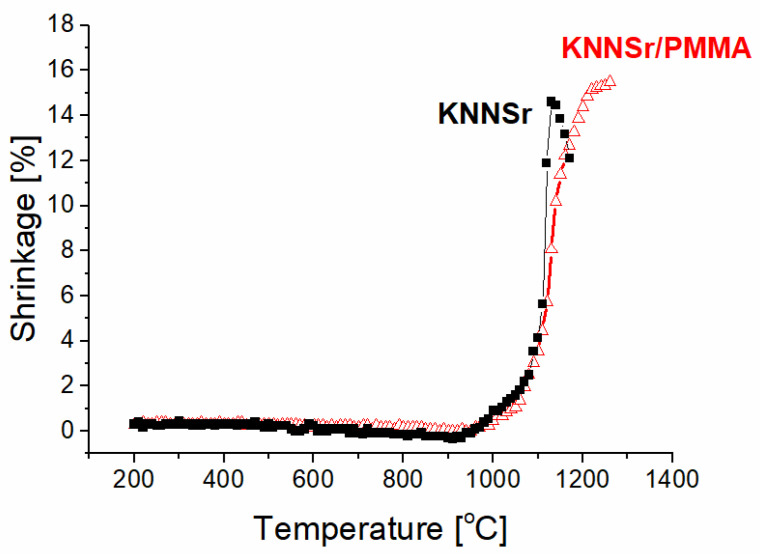
Shrinkage–temperature curve for KNNSr and KNNSr/PMMA. The KNNSr/PMMA powder compact was preheated at 350 °C.

The sintering behaviour of KNNSr was investigated using optical dilatometry. We measured the linear shrinkage as a function of temperature for the KNNSr powder compact and for the KNNSr/PMMA powder compact, with the latter preheated at 350 °C (Figure 3). Both powders begin to shrink at 1000 °C. The KNNSr powder reached the highest shrinkage of 14.5% at 1140 °C, where it began to melt. The preheated KNNSr/PMMA follows the dynamic sintering curve of KNNSr up to 1100 °C. It shrinks more slowly at higher temperatures, although it reaches a larger shrinkage of 15.5% at 1200 °C. This behaviour could be related to the lower initial density of KNNSr/PMMA powder compacts compared to KNNSr. Based on the dynamic sintering curve of KNNSr/PMMA powder compacts, we selected 1100 °C as the sintering temperature for processing the porous ceramic.

**Figure 4:**
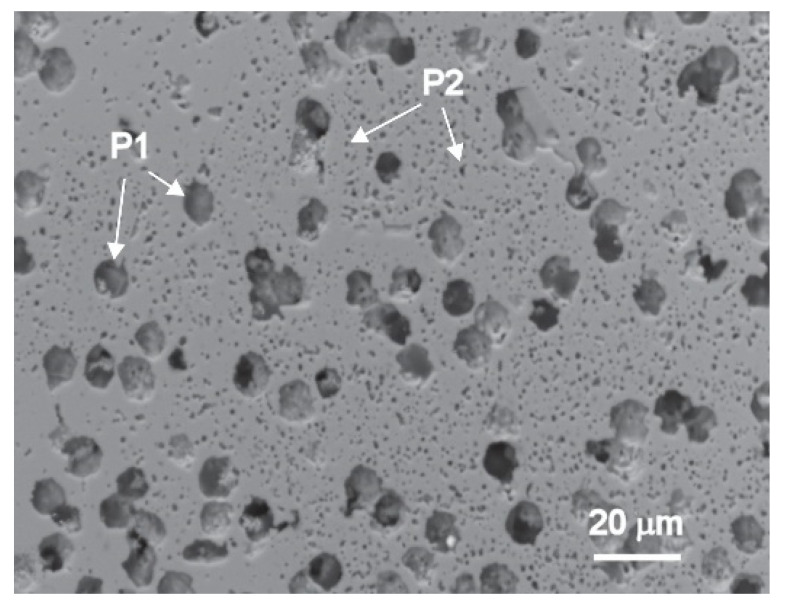
Polished cross-section SEM image of the KNNSr-C ceramic sintered at 1100 °C for 2 h in oxygen.

The polished cross-section SEM image of the KNNSr-C ceramic sintered at 1100 °C is shown in Figure 4.

It is evident that the KNNSr-C ceramic contains a matrix phase (KNNSr), with relatively homogeneously distributed pores. The larger, spherical pores with a diameter of ~8 μm (Figure 4, P1) are in good agreement with the size and shape of the PMMA particles. In addition to these pores, the micrometre-sized pores (Figure 4, P2) can also be seen. These are residual pores from the sintering of the KNNSr powder. The relative porosity of the ceramic is 25 ± 1%.

**Figure 5:**
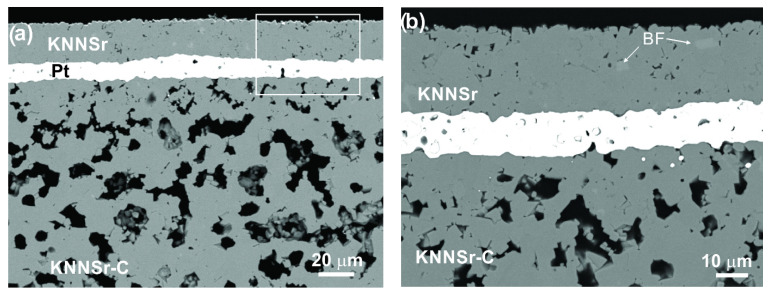
Cross-section SEM/backscattered electron microscopy (BEI) image of KNNSr-T (**a**) and the inset (**b**).

To evaluate the suitability of KNNSr-C ceramics for backing application, their acoustic properties were measured. The ceramic had a longitudinal wave velocity *C_L_* of 4100 m/s and an acoustic impedance of Z = 13.6 MRay, which was derived from *C_L_* and the density of KNNSr-C. The acoustic attenuation coefficient of KNNSr-C measured by a transmission method at a centre frequency of 10 MHz was 0.5 dB/mm/MHz. This value is lower than that of PZT with similar pore size and density, i.e., 1.8 dB/mm/MHz [7]. The main reason for the lower acoustic attenuation coefficient in the case of KNNSr seems to be related to the longitudinal wave velocity. At centre frequency *f_C_* of 10 MHz, the corresponding wavelength *λ =* $\frac{C_{L}}{f_{c}}$ for KNNSr-C is 410 μm. The *C_L_* for PZT-based porous backings is 3400 m/s [7], and the corresponding λ is 340 μm. This means that the λ for the PZT backing is lower than that for KNNSr-C and slightly closer to the pore size (about 8 μm), resulting in a more efficient scattering effect [25] and, consequently, a larger acoustic attenuation. However, the KNNSr-C acoustic attenuation coefficient of 0.5 dB/mm/MHz is sufficiently high to be an efficient backing in high-frequency ultrasonic transducers. To test this hypothesis, we have developed a multilayer structure consisting of a KNNSr thick film on platinised porous KNNSr-C ceramic. The structure is referred to as KNNSr-T. The polished cross-sectional SEM image of the KNNSr-T microstructure is shown in Figure 5.

It can be seen from the images that the KNNSr thick film adhered well to the porous ceramic (KNNSr-C), and we did not observe any delamination. The KNNSr layer was 25 μm thick, with a relatively homogeneous microstructure and a relative density of 97 ± 1%. EDXS analysis (Table 1) showed that the (K+Na)/Nb ratio of the matrix phase, 0.91, was in good agreement with that of the perovskite phase. We also detected a light phase (BF) in the samples with a (K+Na)/Nb ratio of 0.54. The composition of the bright phase corresponds well to the K_5_._75_Nb_10_._85_O_30_, with some Na in the tungsten bronze phase [26]. Note that the amount of Sr is below the detection limit of the method.

**Figure 6:**
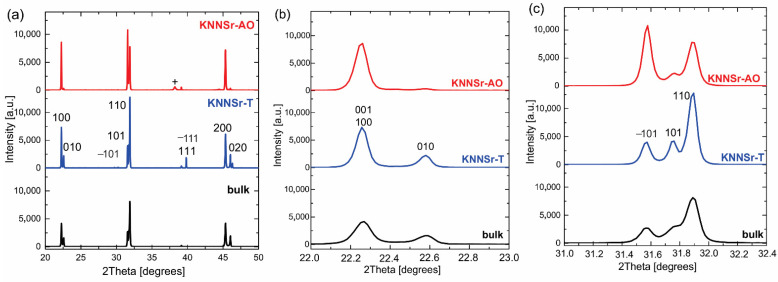
(**a**) X-ray powder diffraction (XRD) patterns of KNNSr-T, KNNSr-AO in KNNSr bulk; (**b**) enlarged view in 2 Theta range 22–23 degrees; (**c**) enlarged view in 2 Theta range 31–32.5 degrees.

**Figure A1:**
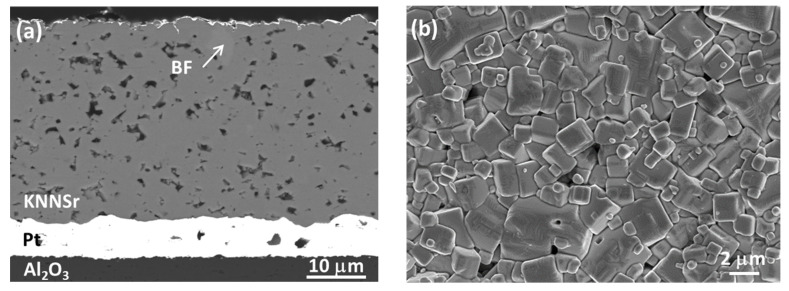
Polished cross-section (**a**) and surface (**b**) SEM images of the KNNSr-AO.

The XRD spectra of the KNNSr-T is shown in Figure 6. All diffraction peaks belong to the perovskite phase indexed with a monoclinic unit cell (PDF 61-0319) [27]. The BF was not detected. Unexpectedly, we found that the intensity of the diffraction peaks corresponds to those of the KNNSr bulk ceramics (bulk). This was not the case for the KNNSr thick films deposited on alumina substrate (KNNSr-AO). The intensities of the (100) and (010) peaks at 2 Theta ~22 degrees and the intensities of the (−101), (101), and (110) peaks at 2 Theta ~32 degrees did not match the intensities for the bulk ceramics (Figure 6b,c). It has been reported that the KNN-based thick films on alumina have a preferential crystallographic orientation in the [100] and [−101] directions, which is related to the stresses developed in the thick films due to the mismatch between the thermal expansion of the KNN and the alumina substrate and the phase transition of the KNN [15,16,28]. Alumina has a thermal expansion coefficient (TEC) of 8 × 10^−6^ K^−1^, while the TEC of KNN is lower, namely, 7.5 × 10^−6^ K^−1^, 4.35 × 10^−6^ K^−1^_,_ and 2.96 × 10^−6^ K^−1^ for the cubic, tetragonal, and monoclinic phases, respectively [29]. The larger shrinkage of alumina compared to KNNSr led to the development of compressive stresses in the KNNSr thick film during the cooling process. The stresses developed in the KNNSr thick film on the KNNSr-C with identical chemical composition are much lower or even negligible due to equal thermal expansion coefficients of the film and substrate. Their release via domain orientation is negligible, and consequently, the KNNSr does not exhibit a preferred crystallographic orientation. Interestingly, the KNNSr thick film of KNNSr-T was denser than that on the alumina substrate processed under identical sintering conditions, i.e., 97 and 90 ± 1%, respectively (see Appendix D, Figure A1). This could be due to the fact that the sintering conditions for the KNNSr thick film on KNNSr-C are less constrained, which could lead to faster densification and denser microstructure of the thin film [30].

The electromechanical properties of KNNSr thick films on porous ceramic (KNNSr-T) and on alumina (KNNSr- AO) were derived from measurements of the complex electrical impedance around the fundamental thickness-mode resonance in air using a KLM equivalent electrical circuit model (Appendix C).

The electromechanical properties listed in Table 2 show that KNNSr-T has the highest *ε*_33_*^S^/ε*_0_ and *c*_33_*^D^,* in accordance with a high *ρ*, which is comparable to those of bulk ceramics. As for the effective thickness coupling factor *k_t_*, the comparison is different. The *k_t_* is related to *e*_33,_ *ε*_33_*^S^*_,_ and *c*_33_*^D^* via the following expression [31]:

(1) $$k_{t}^{2}=\frac{e_{33}^{2}\;}{\varepsilon_{33\;}^{s}\;\cdot \;c_{33}^{D}}$$

**Figure 7:**
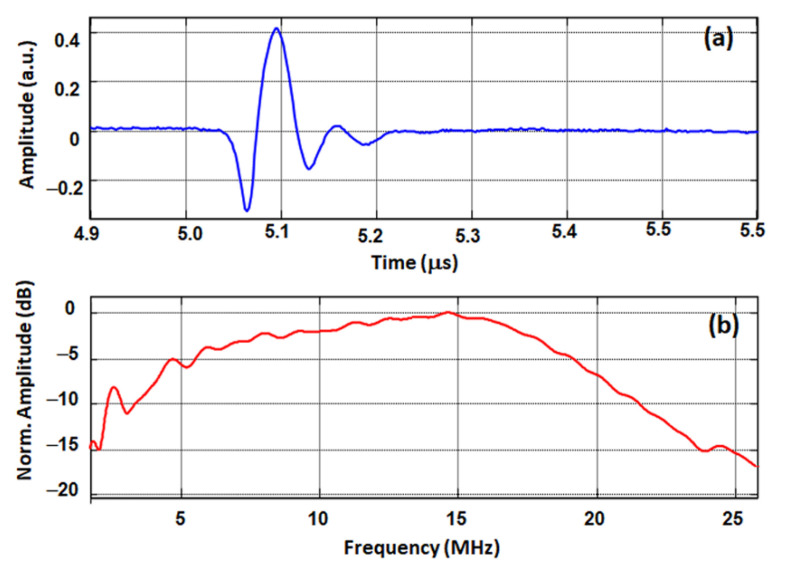
Experimental (**a**) time and (**b**) frequency responses for a KNNSr-T.

Thus, a higher value of *e_33_* and a lower value of *ε*_33_*^S^/ε_0_* and *c*_33_*^D^* eventually lead to a higher *k_t_* for bulk. For KNNSr-T and KNNSr-AO, the e_33_ values are relatively similar, but *ε*_33_*^S^/ε*_0_ and *c*_33_*^D^* are higher for the porous material. This contributes to decrease the *k_t_* value. Finally, the pulse-echo response of KNNSr-T was measured in water at a distance of about 4 mm from a flat metallic target (Figure 7). KNNSr-C with a thickness of 2.2 mm served as an efficient backing and was considered as a semi-infinite medium. We did not detect any back echoes from its rear face, and its attenuation coefficient is sufficiently high at the operating frequency. From this response, the sensitivity S of the test structure was deduced using the following equation:

(2) $$S(\mathrm{dB})=20\cdot log_{10\;}\left(\frac{U_{e}}{U_{r\;}}\right)$$

where *U_e_* and *U_r_* are the excitation and reception peak voltages.

The KNNSr-T had a sensitivity of −60 dB and a centre frequency of 15 MHz. The corresponding fractional bandwidth (BW) was very high, with a value of 127% at −6 dB. Note that for the pulse-echo measurements of the KNNSr-T, the beam was not focused as is the case with other transducers [11,32]. Larger sensitivities were measured for focused-beam transducers, i.e., −31 and −41 dB for KNN and PZT transducers, respectively, but the BWs were lower, i.e., 42 and 93% at −6 dB, respectively. The very wide bandwidth of the KNNSr-T provides a large axial resolution, and, in the case of beam focusing, such a structure shows a high potential for imaging applications.

## 4. Conclusions

In this study, we establish a relationship between the phase composition and microstructure and the functional response of KNNSr thick films on a porous KNNSr ceramic backing and measure the pulse-echo response of the multilayer structure. The KNNSr-C prepared by the organic template method had a homogeneous microstructure with 8 μm-sized spherical pores. The sufficiently high attenuation coefficient of 0.5 dB/mm/MHz indicates that this ceramic is an efficient backing in high-frequency ultrasonic transducers. The 25 μm thick KNNSr on KNNSr-C had a homogeneous microstructure, with a relative density of 97%. This exceptionally high density could be related to the matching thermal expansion coefficients of the thick film and the substrate. Accordingly, the stresses develop at a much lower extent than in KNNSr on alumina, which is reflected in a randomly oriented crystal structure, similar to bulk ceramics. KNNSr on KNNSr-C had a higher dielectric permittivity and a slightly lower *c*_33_*^D^* compared to the bulk ceramic, resulting in a *k_t_* of 29%. The low k_t_ resulted in a relatively low sensitivity of the multilayer structure, i.e., −60 dB. However, the sensitivity in the focal zone can be significantly improved by focusing the beam with a lens. The corresponding fractional bandwidth (BW) of the test structure was very large, 127% at −6 dB. This study shows that completely lead-free multilayer structure of KNNSr thick films integrated on ceramic with identical chemical composition but with tailored porosity and pore size have high potential for ultrasonic transducer imaging applications.

## Figures and Tables

**Table 1:** EDXS analysis of KNNSr thick films.

Atomic Percent					
	Na	K	Sr	Nb	(K+Na)/Nb
KNNSr-nominal composition	10.09	10.09	0.1	20	1.009
Matrix phase	9.5 ± 0.27	9.5 ± 0.15	0	20.9 ± 1.1	0.91
TB-nominal composition	0	12.34	0	23.28	0.530
Light phase	2.7 ± 0.4	9.9 ± 0.02	0	23.4 ± 0.3	0.54

**Table 2:** Electromechanical properties of KNNSr-T and in KNNSr-AO. The literature data on KNNSr bulk are given for comparison.

Thick Film	*ρ* (%)	*ε*_33_ *^S^* */ε*_0_	*k_t_* (%)	*c*_33_*^D^* (GPa)	*e*_33_ (C/m^2^)
KNNSr-T	97	330	29	217.3	7.3
KNNSr-AO	90	160	45	123	6.6
KNNSr bulk [15]	97	300	41	186	9

*ρ*—relative density, *ε*_33_^*S*^/*ε*_0_—dielectric constant at constant strain, *k_t_*—effective thickness coupling factor, *c*_33_^*D*^—elastic constant at constant electric displacement, *e*_33_—piezoelectric coefficient.

## Data Availability

Not applicable.

## Appendix A. Processing of Porous KNNSr Ceramic

The porous KNNSr ceramic (KNNSr-C) acting as a backing, was prepared by sacrificial template method from the KNNSr powder and an organic template, i.e., spherical particles of polymethyl methacrylate with median particle sizes of 10 µm (PMMA) (Soken Chemical & Engineering Co., Tokyo, Japan). KNNSr and PMMA were homogenised in ultrapure water (resistivity of 18 MΩ) using hetero-coagulation technique. 5 vol% of PMMA was electrostatically stabilised in ultrapure water at pH 7. After mixing the suspension in a ball mill for 1 h at 150 min^−1^, its pH was 7.7 and the zeta potential (ZP) of PMMA was −25 mV. Separately, 5 vol% of KNNSr was stabilised in ultrapure water with 6 wt.% of polyethyleneimine (PEI, average molecular weight of 10,000, Alfa Aesar, Karlsruhe, Germany) with respect to the solid load. The suspension mixed in a ball mill for 1 h at 150 min^−1^ has pH of 11.7 and the ZP of KNNSr was +40 mV.

The both suspensions were mixed together in the volume ratio KNNSr: PMMA of 70:30 and homogenised in a ball mill for 1 h at 150 min^−1^. The suspension was dried at 105 °C. Powder compacts with diameter of 12 mm and height of 8 mm were isostatically pressed at 50 MPa and afterwards preheated at 500 °C for 2 h in the flow of a synthetic air. The heating rate was 1 °C/min. Then, the samples were sintered at 1120 °C for 2 h in a flow of synthetic air with heating and cooling rates of 5 °C/min.

## Appendix B. Acoustical Properties

Acoustic properties, i.e., longitudinal wave velocity and attenuation of porous KNNSr ceramic were measured using a transmission method with two commercial transducers (one transmitter and one receiver) with a centre frequency (*f_c_*) at 10 MHz (Olympus, Rungis, France). The experimental setup consisted of a pulser/receiver generator (Olympus, Panametric 5900 PR, Rungis, France) and an oscilloscope (LeCroy waverunner 64XI, Charlottesville, VA, USA) to visualise and save the data. The sample used was the cylinder-shape KNNSr-C (without electrode). Before the measurements, a thin parylene layer (PDS2000, SCS, Indianapolis, IN, USA) was deposited on all the surfaces to prevents the KNNSr from humidity. The corresponding uniform thickness is lower than 100 nm and is negligible for the acoustic measurements. The time-of-flight (TOF) and amplitude of the transmitted ultrasonic signals were registered. According to the TOF value and thickness of the sample, the longitudinal wave velocity was deduced. Two successive measurements were performed with the same sample, first with an initial thickness of 5.8 mm and the second, after thinning the sample to 4.3 mm. The attenuation coefficient *α* was deduced with the following relation:

(A1) $$\alpha =\frac{A}{f_{c}\;t}\;$$

where *A* is the logarithmic difference in amplitude between the two transmitted signals (for the two thicknesses) expressed in dB, *f_c_* is the centre frequency of the transducers used and t is the difference of the thickness between the two samples (in our case 1.5 mm). α is expressed in dB/mm/MHz where a linear behaviour is assumed in the studied frequency range around 10 MHz.

## Appendix C. Electromechanical Properties

The electromechanical properties of KNNSr-T were deduced from the measurements of the complex electrical impedance around the fundamental thickness-mode resonance in air. The experimental set-up was composed of an HP4395A spectrum analyser (Agilent Technologies Inc., Palo Alto, CA) and its impedance test kit. An equivalent electrical circuit (KLM, [21]) was used to calculate the theoretical complex electrical impedance of multilayer structures with several inert layers and one piezoelectric layer (screen-printed thick film). For KNNSr-AO inert layers are alumina substrate, platinum bottom electrode and gold top electrode. For the calculation, thickness and acoustic properties (acoustic attenuation, acoustic impedance and velocity) are taken into account [33]. For KNNSr-T sample, acoustic properties of the KNNSr-C previously measured were used and also those of platinum bottom and top gold electrodes. All these data were considered as constants in a fitting process to deduced thickness-mode parameters such as the effective thickness coupling factor (k_t_,) the longitudinal wave velocity (*C_L_*) and the dielectric constant at constant strain (*ε*_33_^*S*^/ε_0_) [34,35]. According to these extracted parameters, the piezoelectric coefficient e_33_ and the elastic constant (*c*_33_*^D^*) were also deduced [31].

## Appendix D. KNNSr Thick Films on Al2O3 (KNNSr AO)

Polished cross-section and surface SEM images of the KNNSr-AO is shown in Figure A1.

The KNNSr thick film is well adhered to the substrate. We have not detected any secondary phase at the Al_2_O_3_/Pt/KNNSr interfaces meaning that the KNNSr and the substrate are chemically compatible phases under selected sintering conditions.

The as-deposited layers (3 passes) had a thickness of 57 μm. After sintering the KNNSr-AO had thicknesses of 39 μm, meaning that the KNNSr shrunk during the sintering in thickness direction for 31%. The relative density, calculated from the SEM images, was 90 ± 1%.

The microstructures of the sample were relatively homogeneous and we have not observed any macro defects, such as cracks. By EDXS analysis we conformed that the matrix phase corresponds to the perovskite phase with (K+Na)/Nb ratio of ~1. We detected also a TB, bright phase. The grain size of KNNSr-AO ranged from ~0.4 μm to ~6 μm.
